# Lignosulfonate Microcapsules for Delivery and Controlled Release of Thymol and Derivatives

**DOI:** 10.3390/molecules25040866

**Published:** 2020-02-16

**Authors:** Claudio Piombino, Heiko Lange, Federica Sabuzi, Pierluca Galloni, Valeria Conte, Claudia Crestini

**Affiliations:** 1 Department of Chemical Science and Technologies, University of Rome ‘Tor Vergata’, Via della Ricerca Scientifica, 00133 Rome, Italy; piombino.cla@gmail.com (C.P.); galloni@scienze.uniroma2.it (P.G.); valeria.conte@uniroma2.it (V.C.); 2 Department of Pharmacy, University of Naples ‘Federico II’, Via Domenico Montesano 49, 80131 Naples, Italy; 3 Department of Molecular Science and Nanosystems, University of Venice Ca’ Foscari, Via Torino 155, 30170 Venice Mestre, Italy

**Keywords:** lignosulfonate, thymol, microcapsules, ultra-sonication, encapsulation efficiency, release kinetics

## Abstract

Thymol and the corresponding brominated derivatives constitute important biological active molecules as antibacterial, antioxidant, antifungal, and antiparasitic agents. However, their application is often limited, because their pronounced fragrance, their poor solubility in water, and their high volatility. The encapsulation of different thymol derivatives into biocompatible lignin-microcapsules is presented as a synergy-delivering remedy. The adoption of lignosulfonate as an encapsulating material possessing relevant antioxidant activity, as well as general biocompatibility allows for the development of new materials that are suitable for the application in various fields, especially cosmesis. To this purpose, lignin microcapsules containing thymol, 4-bromothymol, 2,4-dibromothymol, and the corresponding *O*-methylated derivatives have been efficiently prepared through a sustainable ultrasonication procedure. Actives could be efficiently encapsulated with efficiencies of up to 50%. To evaluate the applicability of such systems for topical purposes, controlled release experiments have been performed in acetate buffer at pH 5.4, to simulate skin pH: all of the capsules show a slow release of actives, which is strongly determined by their inherent lipophilicity.

## 1. Introduction

The increasing number of antibiotic resistant pathogens has prompted the development of improved and innovative systems with antibacterial properties, without toxic effects on human cells [1]. In recent years, research in the biopharmaceutical sector has especially focused on the combination of natural-derived elements, aiming at the development of biocompatible controlled-release systems. Controlled drug delivery and release offers numerous therapeutic benefits in terms of pharmacokinetics and pharmacodynamics, while also avoiding formulation problems that are usually linked to the hydrophobicity of many active ingredients [2]. In this perspective, the biocompatibility of the drug vehicle is pivotal [3,4]. In this scenario, entrapment, in various forms of actives in matrices made of natural polymers, represents an interesting approach; numerous efforts have been published in this direction, using practically every type of natural polymer physically and chemically suitable [5]. Lignin, in this respect, possesses key features to be part of this gathering, as has been recently summarized [6].

**Figure 1:**
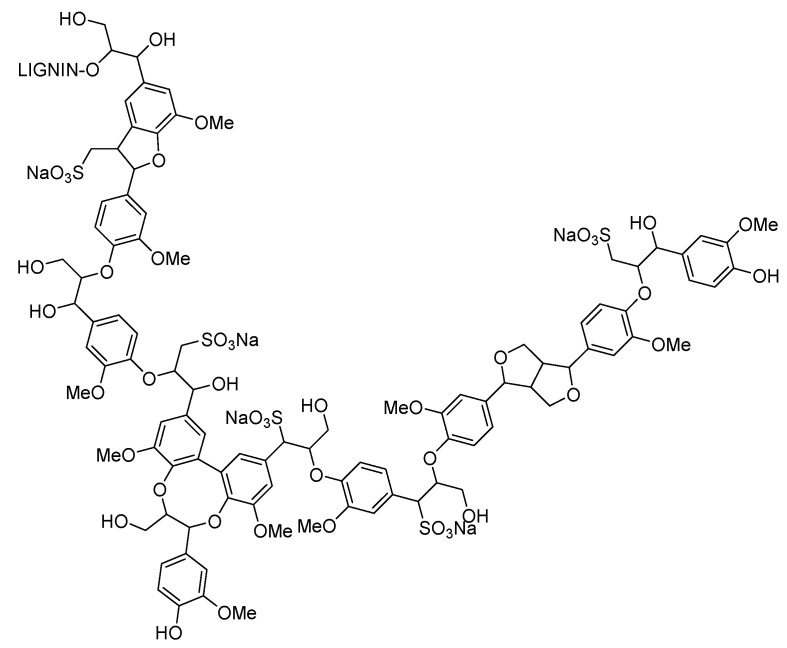
Generic structure of softwood lignosulfonate (**SLS**) oligomers used before in softwood lignosulfonate-based microcapsules (**SLS-MC**)-generation [12].

Lignin as abundant plant polymer is considered the most eco-friendly, cheap, and renewable source of aromatic compounds for the chemical industry, for various applications in the fields of performance materials and for use in personal care products as well as cosmetics, including functional cosmetics [7,8]. Lignins generally contain polar hydrophilic aliphatic and phenolic OH-groups in a rather lipophilic arylpropionic backbone [9]. However, lignin structures exhibit always plant-specific and isolation process-depending differences: as a function of the isolation process, fragmentations or chemical functionalization of the polymer are observed [10,11]. Lignosulfonate (**LS**) is a type of lignin that is isolated treating wood biomass with metal sulfites and sulfur dioxide in an acidic environment. The harsh reaction conditions cause lignosulfonates to display both the typical aliphatic and phenolic OH-groups, as well as sulfonate moieties. Figure 1 shows a generic lignosulfonate structure.

The mix of functional groups renders typical **LSs** water soluble over a wide pH range [12,13]. Moreover, **LSs** possess a very interesting toxicity profile towards plants, animals, and humans, and they are consequently used as technological additives, for example, as emulsifier in animal feeds [14,15]. Likewise, lignosulphonate is not an irritant or sensitizer to skin and eyes [16], therefore applications in cosmetics are currently allowed.

Encapsulation by sonication is a recently developed technique for the synthesis of natural polyphenols microcapsules that requires minimum amounts of time for preparation and only generates minimum amounts of aqueous waste [6,8,12,17]. The energy evolved during sonication can, in fact, trigger supramolecular stacking interaction in natural polyphenolic macromolecules, such as tannins or lignins, most noteworthy also without the addition of crosslinking, and thus eventually only additionally stabilizing agents [18,19,20]. This allowed for the generation of different families of capsules for controlled stimuli responsive active release under different conditions [6,8,17,21,22]. Tuning of the sonication power allows for either simple stacking or, under higher power treatments, crosslinks between the macromolecule chains to generate thicker capsules. Such materials show high stability, not only in the isolated form, but also in cosmetic media and, therefore, are interesting vectors for innovative formulation in health, personal care, and cosmetics. The structural features of **LSs** also render them optimal shell components for water-dispersible nano- and microcapsules that can be generated easily while using ultrasonication and are suitable for hosting rather water-insoluble actives [6,23].

Thymol (**T**) is a natural-derived monoterpenoid phenolic substrate. It is the main monoterpene phenol occurring in essential oils isolated from plants belonging to the Lamiaceae family [24]. Thymol is widely known in the chemical industry, because of its insecticidal [25,26], antibiotic [24,27], antitubercular [28], antileishmanial [29], and antineoplastic [30] properties, besides a general non-genotoxicity or cytotoxicity on human cells [31,32,33]. However, in the last few years, the interest is moving toward brominated thymol derivatives [34], which showed enhanced bioactivity against different bacterial and fungal strains [35,36]. In particular, 4-bromothymol (**BT**) was recently discovered to have an antimicrobial action up to 15 times higher than thymol itself, which would make it a remarkable substrate for applications in the health care field [36]. Additionally, as phenolic compounds, thymol derivatives exhibit high antioxidant properties; therefore, they can find application in the field of disinfectant formulations. To overcome the low water solubility, thymol has entrapped into biocompatible structures, such as cyclodextrins [37] or other natural polymers, like tragacanth gum or chitosan [38]. Most importantly, activities were not found to be altered upon microencapsulation in natural polymers [39].

In light of the growing interest in such area, we embarked to develop an efficient and biocompatible delivery system, showing high antimicrobial and antioxidant activity, to be adopted for topical applications in cosmesis. Therefore, in the present work, we illustrate a simple and efficient method for the encapsulation of thymol and its derivatives in lignosulfonate-based microcapsules. Specifically, the combination of a phenolic active with a polyphenolic matrix is, in principle, suitable for enhancing overall effectiveness of the system on the basis of expectable synergistic effects [40,41]. The idea is using an oligo- and polymeric shell system that itself can function as antioxidant, to protect the thymol or thymol derivative as a low molecular weight mobile antioxidant and antimicrobial agent. Together with thymol (**T**), five derivatives were synthesized and encapsulated, namely 4-bromothymol (**BT**), 2,4-dibromothymol (**DBT**), as well as the corresponding methylated derivatives, *o*-methylated thymol (**MT**), *o*-methylated bromothymol (**MBT**), and *o*-methylated dibromothymol (**MDBT**). Morphology and characterising statistical data were evaluated in order to exhaustively describe this novel system for an eventual application in the biomedical area. Additionally, encapsulation efficiencies and release behaviours were determined.

## 2. Results and Discussion

### 2.1. Green Syntheses of Thymol Derivatives

**Scheme 1:**
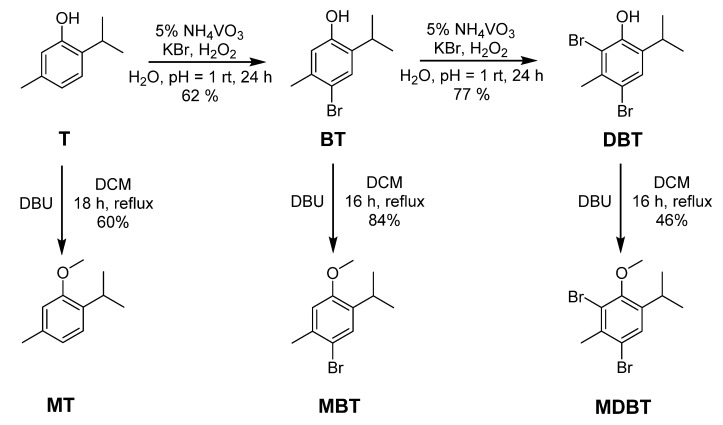
Overview of synthetic efforts leading to the thymol derivatives used in this study.

Mono- and dibromination of thymol was successfully achieved in water, while using a sustainable Vanadium catalysed process that was recently optimised by Conte et coll. [36,42] (Scheme 1).

The two brominated thymol derivatives 4-bromothymol (**BT**) and 2,4-dibromothymol (**DBT**) could be obtained in satisfying yields of 62 and 77%, respectively. Subsequently, the protection of the phenolic OH-group has been performed, while using dimethyl carbonate as a green and safe methylation reagent [43,44]. The reactions proceeded smoothly as well in useful to satisfying yields, between 46 and 84% (Scheme 1). All of the generated thymol derivatives were characterised by ^1^H NMR spectroscopy and mass spectrometry for structural verification and purity control prior to their use as active in softwood lignosulfonate-based microcapsules (**SLS-MCs**).

### 2.2. Generation of Softwood Lignosulfonate Microcapsules Containing Thymol and Derivatives

For the generation of microcapsules from natural polyphenols (**NPs**), sonochemistry proved to be exceptionally useful, as demonstrated in various studies before [8,12,17,21,22]. Capsules were generated according to a protocol previously validated for **SLS [12]** starting from an oil-in-water (o/w) emulsion that was obtained by mixing and vigorously shaking a 1/1-mixture (*v/v*) of the following two solutions: a 5 mg/mL aqueous solution of softwood lignosulfonate (**SLS**) at pH = 12 and a 5 mg/mL solution of thymol derivative in olive oil spiked with 5% (m/m) of linoleic acid (**LA**). The alkaline environment was previously shown to be necessary to both guarantee highly amphiphilic character and full solubility of the oligomeric and polymeric lignins for reliable capsule generation [12]. The oil in water emulsion was then subjected to ultrasonication that promotes microcavity formation. **SLS** molecules move to the oil-water interface, where aggregation is induced by interactions of the aromatic moieties. Such interactions allow for the formation of microcapsules, in which the active component, i.e., thymol or thymol derivatives, remains encapsulated [17]. 

**Figure 2:**
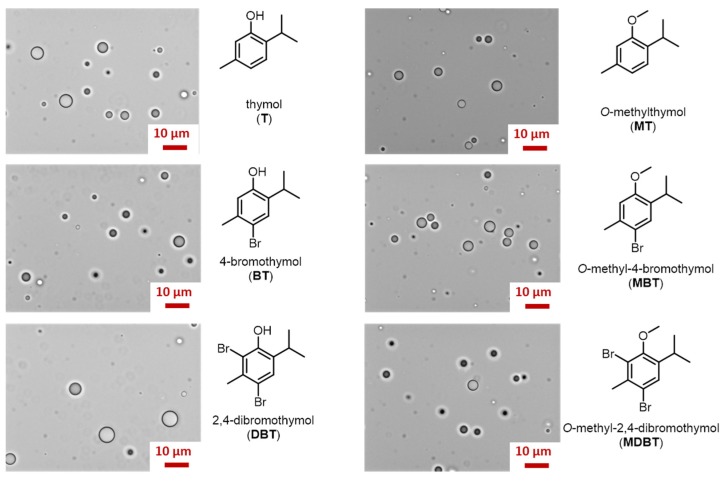
Micrographs of realised **SLS-MCs** containing thymol and derivatives.

**Figure 3:**
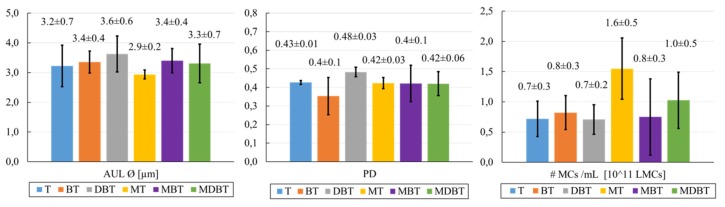
Active-to-active comparison of average upper limits (AUL) of Ø (**left**), polydispersities (PD) (**centre**) and yield (**right**), considering the mean values of three batches, for each parameter. Standard deviations are represented by error bars.

Optical microscopy images confirmed capsule formation for all of the systems under analysis. Figure 2 reports representative images of each system. Only single spherical structures have been observed; capsule aggregation did not occur in the analysed samples. Detailed statistical quantitative and morphological analyses were achieved by combining imageJ elaboration of microscopy images with generation of statistical data; the results are summarised in Table 1, and graphically compared in Figure 3.

The results averaged over three batches indicate a high overall similarity in terms of capsules dimension and yield across the tested actives. All systems under analysis show similar values of averaged upper limits of diameters (AUL Ø) of around 3.2 µm, with comparable polydispersities (PD) between 0.4 and 0.5. The deprotonation of the phenolic OH-group induced by the basic aqueous solution does not interfere with the effectiveness of the biphasic system, which seems to be entirely controlled by the physical parameters and the immiscibility of the oil and the water. The observed low variability in terms of morphological aspects is in line with observations that were made before on a series of other polyphenol-based systems facultatively containing actives [17].

Effects that are eventually attributable to the physical-chemical differences of thymol derivatives are observed in the yield of **MCs** obtained for the different systems (Figure 3). In particular, yields for methylated derivatives, i.e., **MT**, **MBT**, and **MDBT**, are, by tendency, higher than the ones found for free phenols, peaking in the **MT_SLS-MC** system. This finding can be explained on the basis of the structural differences and the resulting changes in physical-chemical properties. This affects not only solubility and, thus, distribution of the actives between the oil and the water phase, but also the possible types of electronic interactions the actives can be undergone with the lignosulfonate shell material on the basis of the aromatic moieties present in the systems. An overview of such interactions and the effects of various conditions on their stability have also been discussed before for lignosulfonate capsules [12]. The lignosulfonate is principally capable of acting as surfactant, a fact exploited since decades by now [45,46,47]; chosen conditions adapted from earlier studies [12] represent optimised conditions in the absence of an active. It could be speculated that actives that are reactive under the chosen basic conditions, i.e., free phenolics **T**, **BT,** and **DBT**, can interfere with the surfactant character, rendering the oil-in-water emulsion less stable by increasing surfactant strength in the overall system. Such an effect would be absent in the methylated systems. Thus, the amount of oil would be crucial.

**Figure 4:**
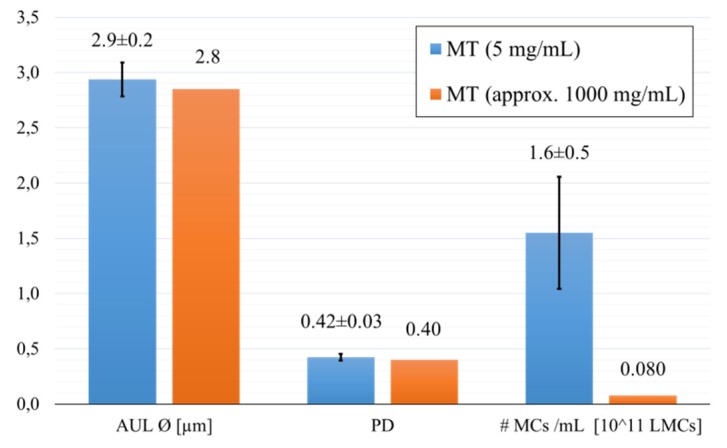
Graphical comparison of the results obtained for different **MT** concentrations in the **MT_SLS-MC** system.

To test this hypothesis, two control experiments were performed: it is, in principle, possible to generate capsules on a biphasic system, in which the neat active forms the hydrophobic phase as counterpart for the aqueous polyphenol phase. While **T** and **BT** are solids, **DBT**, **MT**, **MBT**, and **MDBT** represent oils at room temperature and standard pressure, being suitable for an oil-free application. *o*-methylated thymol (**MT**) has been chosen for a proof of concept. Interestingly, an emulsion indicating successful capsule formation was not formed. Probably, this has to be explained by the strong surfactant character of **SLS** that solubilizes **MT**, being eventually enhanced with ultrasonication. In light of these results, a 1:1 (v/v) ratio of active and olive oil was tested as oily phase. After initial mixing, ultrasonication and standard **SLS-MC** isolation via centrifugation, only a thin foamy layer was observed. The results are compared to the ‘standard’ **MT_SLS-MC** system in Figure 4.

Independent of the **MT** concentration in the active phase, the mean diameter and polydispersion index exhibit similar values. The data are further comparable to **SLS-MCs** produced before, and analogous, in general, to the polyphenol-based capsules generated via the ultrasonication approach. This finding underlines that the capsule dimensions are solely controlled by the physical parameters of the generation set-up, supporting the general applicability of the method for producing various capsule systems within biomedical useful dimensions. However, the oil to water ratio has a significant effect on the yield of capsules. A lower oil to water ratio, corresponding to a non-buffering of the surfactant capability of **SLS**, reduces the yield by about twenty times. This highlights the importance of the stability of the initial emulsion and the sensitivity of the capsule formation process to an eventually present inherent surfactant character of the polyphenolic shell material.

### 2.3. Encapsulation Efficiencies

The phenolic nature of the actives and, thus, their similarity to the shell-forming structures might interfere with the capsule formation, as well as cause variations in encapsulation efficiencies and/or nature. However, polyphenol-based systems studied so far mixed well with the oily phase, while showing little to no tendencies for the aqueous solution that did not contain the shell material [8,17,21,22]. Thus, this system is different in two respects. Thymol (**T**) and its phenolic brominated derivatives, **BT** and **DBT**, are expected to enter in an equilibrium between deprotonated and protonated stage at pH 12, with the salts being more water soluble and effectively reducing the amount of active in the oily phase. The strong surfactant character of the shell material, i.e., **SLS**, is additionally expected to drive active molecules into the aqueous phase. Overall encapsulation efficiency has been evaluated by three different measurements for each system: (i) directly for determining the amount of active entrapped within the oily core; (ii) indirectly for determining how much active was left over in the aqueous waste phase after capsules isolation; and, (iii) in a capsule-washing while using hexane for determining how much active is relatively loosely bound to capsule surfaces. The amount of actives that is eventually entrapped within the shell, i.e., within layers of **SLS** molecules, has not been evaluated, since the shells were expected to be very thin based on previous analyses of similar systems [17] and, thus, hosting practically only trace quantities of actives.

**Figure 5:**
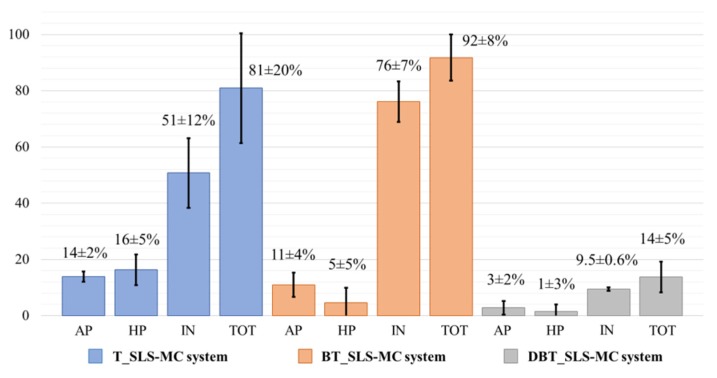
Comparison of quantities of active in aqueous phase (AP), hexane phase (HP) corresponding to surface-bound active (SBA), inside capsules (IN) and overall sum (TOT) for **SLS-MCs** containing unprotected thymol and derivatives. Data are presented as percentage ± standard deviation over three batches.

Encapsulation efficiency (EE) data, as listed in Table 1 and graphically displayed in Figure 5, indicate that unprotected thymol derivatives can be satisfyingly encapsulated. **T** and **BT** systems show better values with respect to the **DBT** one. In particular, capsules containing the mono-brominated active show the best direct EE value among the free phenolic actives.

A remarkable difference is clearly the relative scarce encapsulation that was observed for the **DBT** system, which, in light of its overall lowest hydrophilicity (log*P* = 5.5), is noteworthy. Stability studies on **DBT** itself in acidic environments and under ultrasonication conditions were performed to test whether the low values are indicative of a decomposition upon ultrasonication, but it resulted in being stable under the conditions tested, so that low total recovery values must be explained differently. Partial reaction with the lignin oligomers under the tested conditions could occur, given the activated aromatic system of **DBT**; this aspect, however, has not been further investigated in this study at this point.

**Figure 6:**
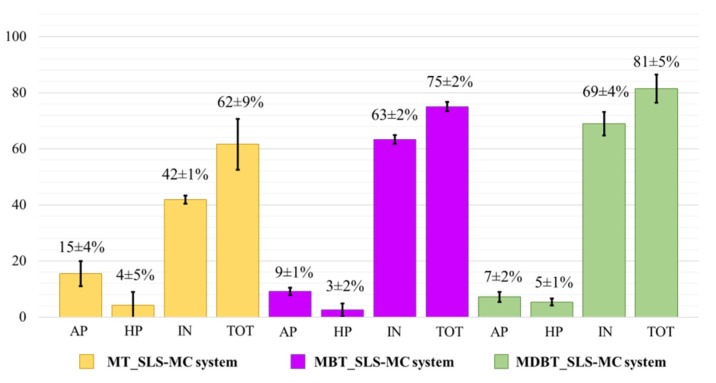
Comparison of quantity of active in aqueous phase (AP), hexane phase (HP) corresponding to surface-bound active (SBA), inside capsules (IN) and overall sum (TOT) for **SLS-MCs** containing protected thymol and derivatives. Data are presented as percentage ± standard deviation over three batches.

The encapsulation of non-phenolic thymol derivatives proceeded by and large smoothly as per direct encapsulation analyses (Table 1, Figure 6). For brominated compounds, **MBT** and **MDBT** found that EEs were always greater than 60%. While considering that percentage of active outside the capsule did not exceed 15% of the starting amount, the encapsulation procedures proved to perform well in the case of most hydrophobic **MBT** and **MDBT** systems. Conversely, direct EE value for **MT** was significantly lower, albeit acceptable, with about 40% for all three batches.

The overall results confirm that encapsulation efficiency directly depend on hydrophobicity, within the standardised theory for formation of polyphenol microcapsules, since the main difference between these systems lies in the more pronounced hydrophobic character in case of the brominated actives [8,12,17].

All of the systems display good encapsulation values, generally around 50% and higher, with exception of the **MT** and **DBT** system. Mono-bromination gave the best results in terms of percentage, but also with respect to reproducibility—the lowest values of standard deviation were found with the mono-brominated microcapsules systems. This suggests that **BT** and **MBT** are reliable substrates within this sequence of procedures. For all thymol derivatives, the amounts of active estimated outside of the capsule were under 20%, with the exception of **T**, for which this value was found to be about 30%. It is evident that estimated active contents data are not summing up to around 100% for all six systems. Noteworthy, for microcapsules containing **T** and **BT**, i.e., for the two most interesting standard systems, the lack of 100% is within the experimental error. For those systems that repeatedly do not arrive at 100%, the loss of active has to be further investigated. Plausible causes for the observed net losses must comprise background reactions and unspecific binding of actives to walls of used reaction vessels since stability issues could be practically excluded (vide supra); however, a more detailed investigation on the cause for the observed loss was not performed at this stage.

### 2.4. Controlled Release Studies

**Figure 7:**
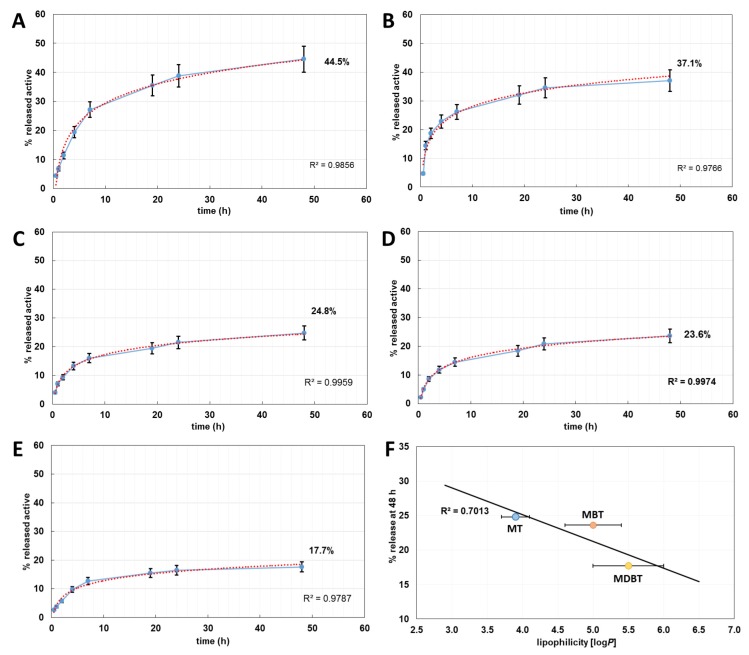
Release kinetics of various thymol containing softwood lignosulfonate microcapsules: (**A**) **T_SLS-MCs**; (**B**) **BT_SLS-MCs**; (**C**) **MT_SLS-MCs**; (**D**) **MBT_SLS-MCs**; (**E**) **MDBT_SLS-MCs**; (**F**) correlation of relative amounts of released actives to lipophilicity of actives. For (**A**) to (**E**), a maximum standard deviation of 10% has been estimated. Regression curves were generated assuming first order kinetics.

Thymol-containing lignosulfonate capsules were thought for use in topical applications in cosmesis. In an attempt to mimic the surroundings of a potential topical application of a **SLS-MC** formulation the human skin, the capsules were added to a 0.1 m acetate buffer at pH = 5.4; earlier studies on **SLS-MCs** have shown that variation in pH is a potent means to trigger **SLS-MC** decomposition [12]. Systems exhibiting satisfying EEs were studied for their release properties; consequently, the **DBT** system has not been tested. At least one release study for each tested active was carried out. Relative amounts of released active were determined on the basis of the same calibration curves that were used for determining the encapsulation efficiency. Room temperature was used, by default, for the release experiments, even though the average skin temperature is 34 °C. Based on initial stability studies performed earlier regarding the stability of **SLS-MCs** [12], the effect of pH and salinity on capsule stability and, thus, release kinetics was considered to be the most important. The release was monitored over a period of 48 h while using a procedure optimised on the basis of the **T_SLS-MC** system that accounts for the solubility characteristics of the encapsulated actives. Figure 7 provides the release profiles for the tested systems.

Release observed for thymol-containing **SLS-MCs** follows a first order kinetic (Figure 7A), reaching 45% of active released after 48 h; at this time, capsules were no longer visually detectable. Release observed for the **BT_SLS-MC** system follows also first order kinetics (Figure 7B), with a total of 37% of **BT** released after the two-day period. The lower amount of released active in the case of **BT** can be explained with the higher hydrophobicity of the active. Similarly, first order kinetic profiles have been obtained for methylated thymol derivatives, although exhibiting smaller initial rate constants. The amounts of active released after 48 h were about 40% lower than those of free phenols because of the increased hydrophobicity. The release characteristics can be roughly correlated to the hydrophobicity of the compounds in the case of *o*-methylated thymol derivatives **MT**, **MBT**, and **MDBT**, as indicated in Figure 7F.

Related studies on polyphenol-based microcapsule systems suggest that the release characteristics can be eventually influenced by manipulating chemically the interactions between the shell-forming oligomers, for example, by introducing cross-linking [6]. Such derivatization of the system has not been targeted in here since this study was designed to shed especially light on the fundamental possibility to encapsulate and release phenolic actives.

## 3. Materials and Methods 

### 3.1. General Information

The solvents and chemical reagents were purchased from Sigma Aldrich/Merck KGaA, Darmstadt, Germany, and they were used without further purifications, if not stated otherwise. Thymol was purchased from Tokyo Chemical Industries Co., Ltd., Tokyo, Japan. Ammonium monovanadate (NH_4_VO_3_) was purchased from Merck KGaA, Darmstadt, Germany. Softwood Lignosulfonate (**SLS**) was obtained from Borregard, Sarpsborg, Norway, and used without further purification. Refined olive oil was spiked with 5% (*w*/*w*) of linoleic acid (LA) for all of the capsule generation experiments. The hydrophobicity of actives was estimated via log***P*** values calculated while using ACDLabs, Toronto, Canada.

### 3.2. ^1^H NMR Spectroscopy

The ^1^H NMR experiments were carried out while using a Bruker, Billerica, MA, USA, 400 MHz Avance III spectrometer that was equipped with a with a 5 mm double resonance broadband BBI inverse probe, operated by Topspin software (Version 3.5). Approximately 5 mg of sample were accurately weighted and dissolved in 600 μL deuterated chloroform (CDCl_3_) and then transferred into 5 mm NMR tubes. All of the spectra were acquired while using eight scans at 27 °C within the standard zg pulse sequence, employing the proton signal of chloroform traces in CDCl_3_ as calibration peak (δ = 7.26 ppm). The NMR data were processed with TopSpin 3.5.7.

### 3.3. Gas Chromatography Coupled to Mass Spectrometry (GC-MS)

A Shimadzu, Kyoto, Japan, GCMS QP2010 Ultra system (gas chromatograph GC2010 Plus, equipped with Shimadzu autosampler AOC-20i) at 70 eV ionization energy was used for all of the analyses. A Supelco, Darmstadt, Germany, fused-silica capillary column SLB-5 ms (30 m long, 0.25 mm thick, 0.25 μm inner diameter) was used as stationary phase, He (UHP grade) as mobile phase; the system was operated in “linear velocity” mode (40 cm/sec) with a starting pressure of 100 kPa, 280 °C injection temperature, and 200 °C interface temperature, running the temperature program: 50 °C start temperature for 1 min, 10 °C min^−1^ heating rate, 280 °C final temperature for 15 min. System control and analyses were realized while using Shimadzu analysis software package Labsolutions-GCMSsolution Version 2.61. The injection volume was 5 uL for each sample.

### 3.4. Synthesis of 4-Bromothymol (***BT***) and 2,4-Dibromothymol (***DBT***)

*4-Bromo-2-isopropyl-5-methylphenol (***BT***).* Following a known literature protocol [42], thymol (**T**) (7.5 g, 50 mmol), and KBr (6.0 g, 50 mmol) were dissolved in a 25 mm aqueous solution of NH_4_VO_3_. Subsequently, H_2_O_2_ (10.0 mL of a 10.4 m aqueous solution, 100 mmol) and HClO_4_ (4.0 mL of a 11.7 m solution, 50 mmol) were added to obtain a pH value of 1. The mixture was vigorously stirred at RT for 24 h. The reaction products were extracted with three portions of diethyl ether. The organic phase was dried over anhydrous Na_2_SO_4_. The solids were filtered off and the solvent was evaporated. The product was purified by a crystallization procedure [36]. The compound was isolated as a white powder. Yield: 62%. Rt: 15.1 min (SLB-5ms). MS (EI, 70 eV): *m/z* = 230 [M^Br81^]^+•^; 228 [M^Br79^]^+•^; 215 [M^Br81^-CH_3_^•^]^+^; 213 [M^Br79^-CH_3_^•^]^+^; 134 [M-CH_3_Br]^+•^. ^1^H-NMR (400 MHz, CDCl_3_): δ = 7.29 (s, 1 H, H-3), 6.64 (s, 1 H, H-6), 3.18–3.07 (hept, 1 H, -C*H*-(CH_3_)_2_, ^3^*J*_C-*H* – (CH3)2_ = 6.9 Hz), 2.30 (s, 3 H, benz-C*H*_3_), 1.23–1.22 (d, 6 H, -CH-(*CH*_3_)_2_, ^3^*J*_C-H – (C*H*3)2_ = 6.9 Hz) ppm.

*2,4-Dibromo-6-isopropyl-3-methylphenol (***DBT***).***DBT** was synthesised starting from 4-bromothymol (**BT**) (2.3 g, 10 mmol) following the procedure for the synthesis of **BT** described above [42]. The crude was purified by a chromatographic column packed with silica, using petroleum ether: dichloromethane 3:1 mixture as eluent (Rf: 0.85). The compound was isolated as a colourless oil. Yield: 77%. Rt: 16.1 min (SLB-5ms). MS (EI, 70 eV): *m/z* = 310 [M^Br81-Br81^]^+•^; 308 [M^Br81-Br79^]^+•^; 306 [M^Br79-Br79^]^+•^; 295 [M^Br81-Br81^ − CH_3_^•^]^+^; 293 [M^Br81-Br79^ − CH_3_^•^]^+^; 291 [M^Br79-Br79^ − CH_3_^•^]^+^; 214 [M^Br81^ − CH_3_Br]^+•^; 212 [M^Br79^ − CH_3_Br]^+•^. ^1^H-NMR (400 MHz, CDCl_3_): δ = 7.31 (s, 1 H, H-5), 5.68 (s, 1 H, O-*H*), 3.31–3.20 (hept, 1 H, -C*H*-(CH_3_)_2_, ^3^*J*_C-*H* – (CH3)2_ = 6.9 Hz), 2.52 (s, 3 H, benz-C*H*_3_), 1.22–1.21 (d, 6 H, -CH-(*CH*_3_)_2_, ^3^*J*_C-H – (C*H*3)2_ = 6.9 Hz) ppm.

### 3.5. Synthesis of Methylated Thymol (***MT***), Methylated Bromothymol (***MBT***) and Methylated Dibromothymol (***MDBT***) [37,38]

*2-Isopropyl-1-methoxy-5-methylbenzene (***MT***).* Thymol (1.0 g, 6.67 mmol) and 1,8-diazabiciclo [5.4.0]undec-7-ene (DBU) (6.67 mmol) were dissolved in 11.0 mL of dimethyl carbonate (DMC). The mixture was vigorously stirred for 16 h under reflux. Afterwards, the crude was re-dissolved in diethyl ether and subsequently washed with an aqueous solution of ammonium chloride (NH_4_Cl, 10% w/v). The organic phase was washed with brine and then dried over anhydrous Na_2_SO_4_. The solids were filtered off and the solvent was evaporated. The crude was purified by chromatographic column packed with silica, while using a gradient of solvent mixture as eluent (from pure petroleum ether to diethyl ether:petroleum ether 9:1 mixture as eluent (Rf: 0.7 in petroleum ether). The compound was isolated as a colourless oil. Yield: 60%. Rt: 10.3 min (SLB-5ms). MS (EI, 70 eV): *m/z* = 164 [M]^+•^; 149 [M − CH_3_^•^]^+^; 134 [M − C_2_H_6_]^+•^; 119 [M − C_3_H_9_]^+•^; 91 [M − C_4_H_9_O^•^]^+^. ^1^H-NMR (400 MHz, CDCl_3_): δ = 7.10–7.08 (d, 1 H, H-3, ^3^*J _H_*_-3 – H-4_ = 7.7 Hz), 6.75-6.73 (d, 1 H, H-4,*^3^J*_H-3 – *H*-4_ = 7.7 Hz), 6.67 (s, 1 H, H-6), 3.81 (s, 3 H, -O-C*H*_3_), 3.32–3.22 (ept, 1 H, -C*H*-(CH_3_)_2_, ^3^*J*_C-*H* – (CH3)2_ = 6.9 Hz), 2.33 (s, 3 H, benz-C*H*_3_), 1.20–1.18 (d, 6 H, -CH-(*CH*_3_)_2_, ^3^*J*_C-H – (C*H*3)2_ = 6.9 Hz) ppm.

*4-Bromo-2-isopropyl-1-methoxy-5-methylbenzene (***MBT***).***MBT** was synthesised starting from 4-bromothymol (**BT**) (1.0 g, 4.50 mmol) following the procedure for the synthesis of **MT** described above [43,44]. The crude was purified by chromatographic column packed with silica, while using a gradient of solvent mixture as eluent (from petroleum ether: diethyl ether 9:1 to pure diethyl ether) (Rf: 0.7 in petroleum ether : diethyl ether 9:1). The compound was isolated as a colourless oil. Yield: 84%. Rt: 14.2 min (SLB-5ms). MS (EI, 70 eV): *m/z* = 244 [M^Br81^]^+•^; 242 [M^Br79^]^+•^; 229 [M^Br81^ − CH_3_^•^]^+^; 227 [M^Br79^ − CH_3_^•^]^+^; 214 [M^Br81^ − C_2_H_6_]^+•^; 212 [M^Br79^ − C_2_H_6_]^+•^; 148 [M − CH_3_Br]^+•^; 133 [M − C_2_H_6_Br^•^]^+^; 118 [M − C_3_H_9_Br^•^]^+^; 91 [M − C_4_H_8_OBr^•^]^+^. ^1^H-NMR (400 MHz, CDCl_3_): δ = 7.29 (s, 1 H, H-3), 6.70 (s, 1 H, H-6), 3.79 (s, 3 H, O-C*H*_3_), 3.28–3.18 (hept, 1 H, -C*H*-(CH_3_)_2_, ^3^*J*_C-*H* – (CH3)2_ = 6.9 Hz), 2.35 (s, 3 H, benz-C*H*_3_), 1.18–1.17 (d, 6 H, -CH-(*CH*_3_)_2_, ^3^*J*_C-H – (C*H*3)2_ = 6.9 Hz) ppm.

*2,4-Dibromo-6-isopropyl-1-methoxy-3-methylbenzene (***MDBT***).***MDBT** was synthesised starting from 2,4-dibromothymol (**DBT**) (0.6236 g, 4.50 mmol) following the procedure for the synthesis of **MT** described above [43,44]. The crude product dissolved in diethyl ether was additionally extracted with an aqueous solution of NaOH (1 m) for ulterior purification, before the organic phase was washed with brine and then dried over anhydrous Na_2_SO_4_. The compound was isolated as a light brown oil. Yield: 46%. Rt: 16.7 min (SLB-5ms). MS (EI, 70 eV): *m/z* = 324 [M^Br81-Br81^]^+•^; 322 [M^Br81-Br79^]^+•^; 320 [M^Br79-Br79^]^+•^; 309 [M^Br81-Br81^ − CH_3_^•^]^+^; 307 [M^Br81-Br79^ − CH_3_^•^]^+^; 305 [M^Br79-Br79^ − CH_3_^•^]^+^; 294 [M^Br81-Br81^ − C_2_H_6_]^+•^; 292 [M^Br81-Br79^ − C_2_H_6_]^+•^; 290 [M^Br79-Br79^ − C_2_H_6_]^+•^; 228 [M^Br81^ − CH_3_Br]^+•^; 226 [M^Br79^ − CH_3_Br]^+•^. ^1^H-NMR (400 MHz, CDCl_3_): δ = 7.38 (s, 1 H, H-5), 3.80 (s, 3 H, O-C*H*_3_), 3.33–3.23 (hept, 1 H, -C*H*-(CH_3_)_2_, ^3^*J*_C-*H* – (CH3)2_ = 6.9 Hz), 2.55 (s, 3 H, benz-C*H*_3_), 1.22–1.20 (d, 6 H, -CH-(*CH*_3_)_2_), ^3^*J*_C-H – (C*H*3)2_ = 6.9 Hz) ppm.

### 3.6. Generation of Lignin Microcapsules (LMCS) by Ultrasonication

Following established procedures [12], the generation of **SLS-MCs** was realized by sonication of an oil-in-water (o/w) emulsion, while applying an acoustic power of 160 W (40% of amplitude) for 10 min at room temperature (RT), using a 3 mm diameter microtip connected to a 20 kHz Branson probe (digital sonifier model 450 L by Branson Ultrasonics, Danbury, CT, USA). A 15 mL centrifuge tube was used as sonication vessel.

### 3.7. General Procedure for the Generation of ***LSL-MCs*** Containing Thymol Derivatives

Following established procedures [12,17], the aqueous solution of **SLS** (5.0 mg/mL) was freshly prepared by dissolving 75.0 mg of **SLS** in 15.0 mL of distilled water. The pH of the system was adjusted to pH = 12.0 by adding dropwise an aqueous solution of NaOH 1 m. The oily solutions containing thymol derivatives were prepared by dissolving 10.0 mg of thymol derivative in 2.0 mL of a solution of 5% (w/w) of linoleic acid in refined olive oil, obtaining a concentration of 5.0 mg/mL. This solution was sonicated in a sonication bath for 1 min and then magnetically stirred for 5 min. The aqueous phase and the oily phase were placed together in the suitable reaction vessel, and the biphasic system was magnetically stirred for 5 min before being ultrasonicated, as described above, for generating **LMCs** in the form of aqueous suspensions. The obtained suspensions were centrifuged at 12,000 rpm for 15 min to separate the foamy capsule-containing phase from the bulk aqueous solution. The foamy phase was washed three times with 1 mL of distilled H_2_O. The different aqueous phases were collected together for further analysis.

### 3.8. Characterization of ***SLS-MCs*** by Optical Microscopy and Statistical Analysis

Isolated **SLS-MCs** were characterised by optical microscopy while using a Zeiss, Oberkochen, Germany, Axio Scope A1 microscope, following an established procedure described in great detail before [12]; statistical analysis on the basis of the images obtained during the microscope analysis, while using the imageJ freeware has been performed for each sample, as described before [12].

### 3.9. Encapsulation Efficiency (EE)

GC-MS analyses using both a direct and an indirect loading study determined the extent of thymol derivatives efficiently incorporated in **SLS-MCs**, as previously established.

*Direct Loading Studies:* A portion of 100 μL of concentrated capsules was added to 1000 μL of ethyl acetate (EtOAc) and to 200 μL of an aqueous solution of HCl (2 m). The mixture was vigorously shaken and centrifuged at 10,000 rpm for 10 min. The organic phase was separated, dried over MgSO_4_, and separated by centrifugation. The percentages of the thymol derivatives contained in the organic phase were determined by gas chromatography coupled to mass spectrometry (GC-MS) analysis on the basis of suitable calibration curves, directly injecting the samples without further dilutions or modifications.

The encapsulation efficiencies are calculated based on the initial amount of thymol derivative used to generate capsules according to:

$$\%\;\mathrm{EE}\;\mathrm{direct}=\frac{\mathrm{Mass}\;\mathrm{of}\;\mathrm{active}\;\mathrm{obtained}\;\mathrm{from}\;\mathrm{direct}\;\mathrm{analysis}}{\mathrm{Mass}\;\mathrm{of}\;\mathrm{initial}\;\mathrm{amount}\;\mathrm{of}\;\mathrm{active}}\times 100$$

Indirect Loading Studies: The aqueous phase separated from the foamy capsule phase during capsule generation was acidified with 2 m aqueous HCl to pH 1–2. 800 μL of aqueous phase were withdrawn and washed with 800 μL of EtOAc. The extraction was repeated for three times. The organic phases were combined, washed with approx. 2 mL of a saturated solution of NaHCO_3_, and then centrifuged at 5000 rpm for 5 min. The organic phase was separated, dried over MgSO_4_, and isolated from the solids centrifuged at 10,000 rpm for 1 min. The percentages of the thymol derivatives contained in the organic phase were determined by GC-MS analysis on the basis of suitable calibration curves, directly injecting the samples without further dilutions or modifications.

The encapsulation efficiencies are calculated based on the initial amount of thymol derivative used to generate capsules according to:

$$\%\;\mathrm{EE}\;\mathrm{indirect}=100-\left[\frac{\mathrm{Mass}\;\mathrm{of}\;\mathrm{active}\;\mathrm{obtained}\;\mathrm{from}\;\mathrm{aq}.\mathrm{phase}\;\mathrm{analysis}}{\mathrm{Mass}\;\mathrm{of}\;\mathrm{initial}\;\mathrm{amount}\;\mathrm{of}\;\mathrm{active}}\times 100\right]$$

Surface bound actives: 100 μL of capsules were added to 850 μL of hexane. The mixture was gently stirred and the organic phase was separated, dried over MgSO_4_, and then centrifuged at 10,000 rpm for 1 min for separating solids and liquid. Percentages of the thymol derivatives contained in the organic phase were determined by GC-MS analysis on the basis of suitable calibration curves, either directly injecting the samples without further dilutions or modifications or after adding 500 μL of a solution of 0.25 mg/mL of the thymol derivative under study before injection.

The encapsulation efficiencies are calculated based on the initial amount of thymol derivative used to generate capsules, according to:

$$\%\;\mathrm{SBA}\;=\frac{\mathrm{Mass}\;\mathrm{of}\;\mathrm{active}\;\mathrm{obtained}\;\mathrm{from}\;\mathrm{hexane}\;\mathrm{phase}\;\mathrm{analysis}}{\mathrm{Mass}\;\mathrm{of}\;\mathrm{initial}\;\mathrm{amount}\;\mathrm{of}\;\mathrm{active}}\times 100$$

### 3.10. Release Kinetics

Starting from literature known protocols, in vitro release of thymol derivatives encapsulated in **SLS-MCs** were performed in acetate buffer 0.1 m at pH = 5.4. Experiments were conducted adding 50 μL of a **SLS-MC** sample to 1000 μL of buffer. This sample was left under very gentle mechanical shaking suitable for preventing capsules from forming a separate layer. At defined time intervals, i.e., 30 min, 1 h, 2 h, 4 h, 7 h, 19 h, 24 h, and 48 h, the buffer phase was withdrawn for analysis, and 1000 μL of fresh buffer were immediately added to the remaining capsules. The buffer that was withdrawn at the designed time point was extracted with 750 μL of EtOAc. 500 μL of the organic phase were isolated, dried over MgSO_4_, and centrifuged. The contents of thymol derivatives were then determined while using a standard addition protocol in the gas chromatography coupled to mass spectrometry (GC-MS)-based analysis: the 500 μL aliquots were diluted with 500 μL of a solution of 0.25 mg/mL of the thymol derivative under study before injection. Quantitative interpretation of every chromatogram was achieved against a standard curve relating chromatographic peak area to the concentration of thymol derivative. The calibration ranges were optimised for each sample as needed.

## 4. Conclusions

A sustainable way towards thymol and thymol derivative-containing lignosulfonate microcapsules, **SLS-MCs**. was presented. In particular, brominated thymol derivatives and corresponding O-methylated compounds were synthesized in acceptable to good yields, through environmental friendly procedures. The synthesized substrates could be successfully incorporated into lignosulfonate-based microcapsules, while using sonication procedures. Single spherical capsules were obtained, with mean diameters of around 3.2–3.4 μm. The encapsulation efficiencies have been measured for each system. The results showed that—with the exception of 2,4-dibromothymol—more than 40% of each substrate was properly encapsulated and, in particular, the mono-brominated thymol derivative (**BT**) shows the best EE value (76%). Release studies have been performed in acetate buffer at pH = 5.4, thus mimicking human skin pH, for a future potential application in cosmetics. All of the studied systems show the first order kinetic, which leads up to 45% of released phenolic actives, while lower release values were observed for *O*-methylated compounds, likely because of the enhanced hydrophobicity.

## Figures and Tables

**Table 1:** Morphological key data for realised systems of **SLS-MCs** containing thymol and derivatives. Data represent the mean values of three different batches.

Entry	Active (log*P*) ^1^	Ø (AUL) ^2^ [µm]	PD ^3^	# ^4^ MCs [10^11^ MCs/mL]	EE ^5^ (Direct) [%]	SB ^6^ Active [%]	Free ^7^ Active [%]
1	**T** (3.3 ± 0.2)	3.2 ± 0.7	0.43 ± 0.01	0.7 ± 0.3	51 ± 12	16 ± 5	14 ± 2
2	**BT** (4.3 ± 0.3)	3.4 ± 0.4	0.4 ± 0.1	0.8 ± 0.3	76 ± 7	5 ± 5	11 ± 4
3	**DBT** (5.2 ± 0.4)	3.6 ± 0.6	0.48 ± 0.03	0.7 ± 0.2	9.5 ± 0.6	1 ± 3	3 ± 2
4	**MT** (3.9 ± 0.2)	2.9 ± 0.2	0.42 ± 0.03	1.6 ± 0.5	42 ± 1	4 ± 5	15 ± 4
5	**MBT** (5.0 ± 0.4)	3.4 ± 0.4	0.4 ± 0.1	0.8 ± 0.6	63 ± 2	3 ± 2	9 ± 1
6	**MDBT** (5.5 ± 0.5)	3.3 ± 0.7	0.42 ± 0.06	1.0 ± 0.5	69 ± 4	5 ± 1	7 ± 2

^1^ calculated log*P* values; ^2^ average upper limit of diameters as detected based on optical micrographs; ^3^ polydispersity; ^4^ number of **SLS-MCs**; ^5^ encapsulation efficiency directly determined; ^6^ surface bound active; ^7^ active in washing solution after **SLS-MC** formation.
